# Mechanical validation of 3D-printed orthopaedic surrogate models for screw insertion training

**DOI:** 10.1007/s00590-026-04766-2

**Published:** 2026-05-11

**Authors:** Austin Thomassen, Benjamin Lee, Mason Granger, Patrick Massey, Brad Chauvin, Francesco Addevico, Giovanni Solitro

**Affiliations:** 1 Department of Orthopaedic Surgery, Louisiana State University Health Shreveport, Shreveport, USA; 2 Ospedale Niguarda Ca’ Granda, Milan, Italy

**Keywords:** training, surrogates, screws, stripping, torque

## Abstract

**Background:**

Screw stripping is a common complication in orthopaedic surgery that can compromise fixation stability. This study evaluates the relationship between stripping torque (ST) and infill density in 3D-printed bone surrogates, establishes a conversion factor between surrogates and ASTM F-1839-08 foam, and tests the hypothesis that screw insertion angle up to 45 °C does not affect ST.

**Methods:**

PLA surrogates with 3D Honeycomb or Gyroid infill patterns were printed at densities ranging from 5 to 27%. These samples were used for screw insertion at angles of 0°, 15°, 30°, and 45°. Partially threaded compression screws were manually inserted while continuously recording torque, performing three repetitions for each configuration (*N* = 3). The maximum torque value was defined as the peak value (ST). Mean values were analyzed using linear regression to evaluate the correlation between density and ST, while multivariable linear regression was used to assess the influence of screw insertion angle.

**Results:**

3D Honeycomb ST ranged from 0.23 Nm ± 0.02 (5%) to 3.89 Nm ± 0.14 (27%), and from 0.32 Nm ± 0.06 to 3.68 Nm ± 0.46 for Gyroid. Both patterns showed strong linear correlations between density and ST (R^2^ > 0.98, *p* < 0.01). ST values in 3D-printed surrogates aligned with ASTM foam (0.52 Nm ± 0.08 to 2.65 Nm ± 0.55 for 10–20 PCF). No significant differences were observed among insertion angles (*p* > 0.65).

**Conclusion:**

3D-printed surrogates with 3D Honeycomb or Gyroid infills at up to 27% density, for insertion inclinations up to 45 degrees exhibit predictable, density-dependent mechanical behavior comparable to ASTM polyurethane foams supporting their use for screw insertion training. Screw insertion angles up to 45° have minimal effect on ST, potentially simplifying surrogate manufacturing.

**Clinical relevance:**

These findings support the use of 3D printed surrogates across diverse training scenarios, including those with uncertain screw insertion angles, and highlight their potential to simulate diverse bone densities and pathologies, offering a more realistic and versatile solution than traditional foams. Their predictable mechanical behavior offers a safe, standardized, and accessible training method for orthopaedic residents, with potential to reduce screw stripping and fixation failure in patients.

**Supplementary Information:**

The online version contains supplementary material available at 10.1007/s00590-026-04766-2.

## Introduction

It is estimated that billions of screws are used for surgery yearly, making them the most utilized surgical implant [1]. Screw stripping can occur when the screw loses fixation in bone [2]. Axial screw rotation causes displacement, limited by the screw head. If overtightened beyond the bone’s ultimate strength, the screw spins freely, causing stripping [3, 4]. It is common in fracture fixation with an incidence of up to 45.4% in trabecular bone [5, 6]. In 90.8% of screw stripping cases, surgeons were unaware stripping had occurred, indicating that screws were severely overtightened before stripping was recognized [1, 6]. Once stripping has occurred, the resistance to pull-out strength can be reduced by as much as 82% [7]. There are multiple strategies to resolve the issue [8–10] but all increase the likelihood of surgical complications [2, 8, 11] that include iatrogenic fractures, infection, neurovascular injury, bleeding, and decreased construct strength [12, 13]. The torque at which screw strip, or stripping torque (ST), is associated with pull-out strength and is influenced by multiple factors, such as depth of screw thread and quality of bone [14–16]. Given the high incidence of screw stripping and significant repercussions, it is imperative to provide surgeons with comprehensive training to limit its occurrence [2, 17].

Residents undergo training for 5 years that is heavily based on Halsted’s “see one, do one, teach one” model [18]. Within this frame work, the fundamental skill of screw fixation into bone relies on the surgeon’s subjective tactile sensation to determine the appropriate torque for optimal stability [17, 19]. This skill is taught via a trial-and-error process, that for tibial bone can require 115–312 screws [20]. Training can be conducted on actual patients under the close supervision of a board-certified orthopaedic surgeon surrogate models or on surrogate models [21, 22] for training in a safe and standardized environment [18, 23, 24].

Surgical simulation has been shown to have educational efficacy, and became mandatory in 2013 by the American Board of Orthopaedic Surgery (ABOS) and Accreditation Council for Graduate Medical Education (ACGME) [25]. There are a variety of surgical surrogate models used in teaching screw placement, including cadaveric, osteomimetic, and 3D printed bone models [21, 26]. Cadaveric specimens have been considered the gold standard for their realistic tissue properties and haptic feedback [27, 28]. However, their use is limited by availability, cost, and inability to mimic specific pathological states [29–31].

Osteomimetic materials, such as polyurethane foams or 3D printed specimens, offer several advantages, including accessibility, standardization material properties, and customization [29, 32, 33].

Surgical simulation is effective, but high costs and logistical constraints of cadaveric specimens and commercial models limit accessibility, underscoring the need for cost-effective, scalable alternatives that preserve relevant mechanical behavior [34–39].

Compared with commercially available polyurethane foams, 3D-printed surrogates provide similar visual realism and equivalent haptic feedback, while offering the distinct advantage of virtually unlimited configurability, in contrast to the limited number of geometrical configurations typically available with foam models [40–42]. In a blinded simulation study, Binnie et al. reported improved tactile realism for drilling and Kirschner-wire insertion using 3D-printed bone models compared to foam models, although no significant difference was observed during screw insertion, and differences in thread engagement were noted [34]. These findings suggest perceived realism may vary by task, highlighting the limitations of subjective assessment alone, emphasizing the need for objective biomechanical validation. In addition, 3D-printed models can be produced in-house with low per-unit production costs [43].

Despite these potential advantages, the mechanical behavior of 3D printed bone surrogates, particularly with respect to screw stripping torque, has not been characterized nor directly compared with standardized ASTM F-1839-08 polyurethane foam densities. Furthermore, because 3D-printed infill architectures are inherently anisotropic and aligned with the printing direction, the influence of screw insertion angle relative to infill orientation on ST remains insufficiently understood [44].

The primary aim of this study was to evaluate the relationship between ST and infill density in 3D-printed bone surrogates and to identify a conversion factor between 3D-printed infill densities and ASTM F-1839-08 polyurethane foam densities commonly used in surgical training. Additionally, given that 3D-printed infill structures are generated along the print direction, a secondary aim was to assess the influence of screw insertion angle relative to the infill orientation on stripping torque. We hypothesized a linear relationship between ST and infill density and no significant influence of insertion angle on ST for inclination up to 45 degrees.

## Materials and methods

### Materials and 3D printing techniques

**Fig. 1:**
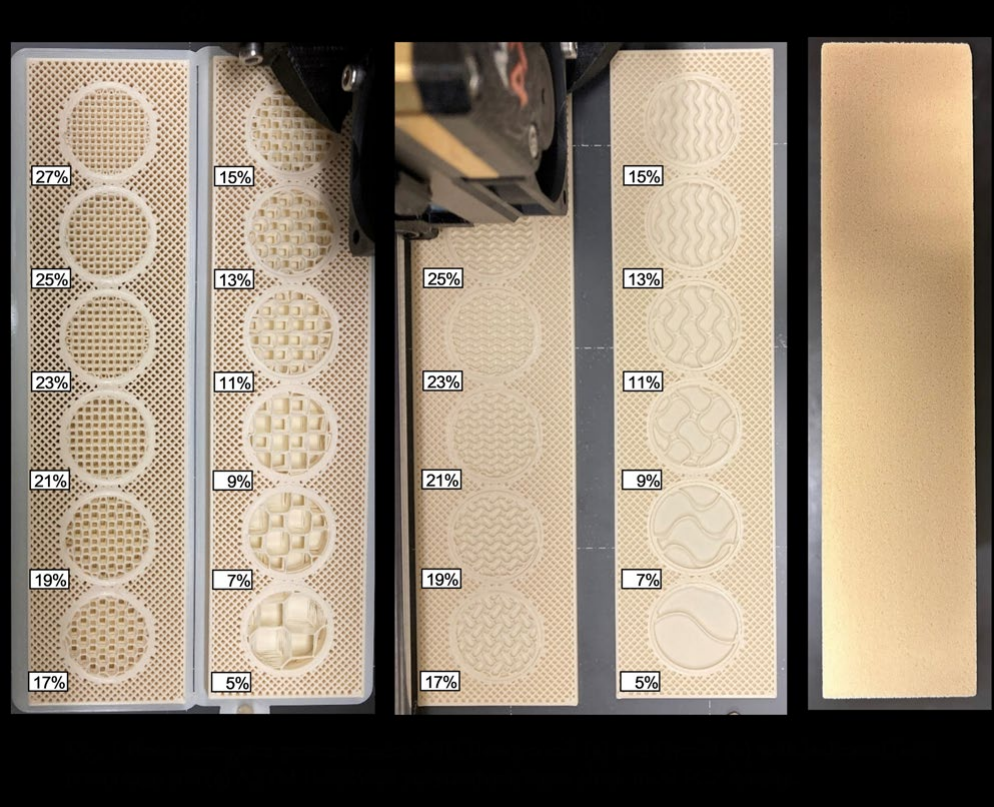
Bone surrogates process made of 3D Honeycomb (**a**) and Gyroid (**b**) with indicated infill percentage and** c** ASTM F-1839-08 polyurethane foam block in 20 PCF density

**Fig. 2:**
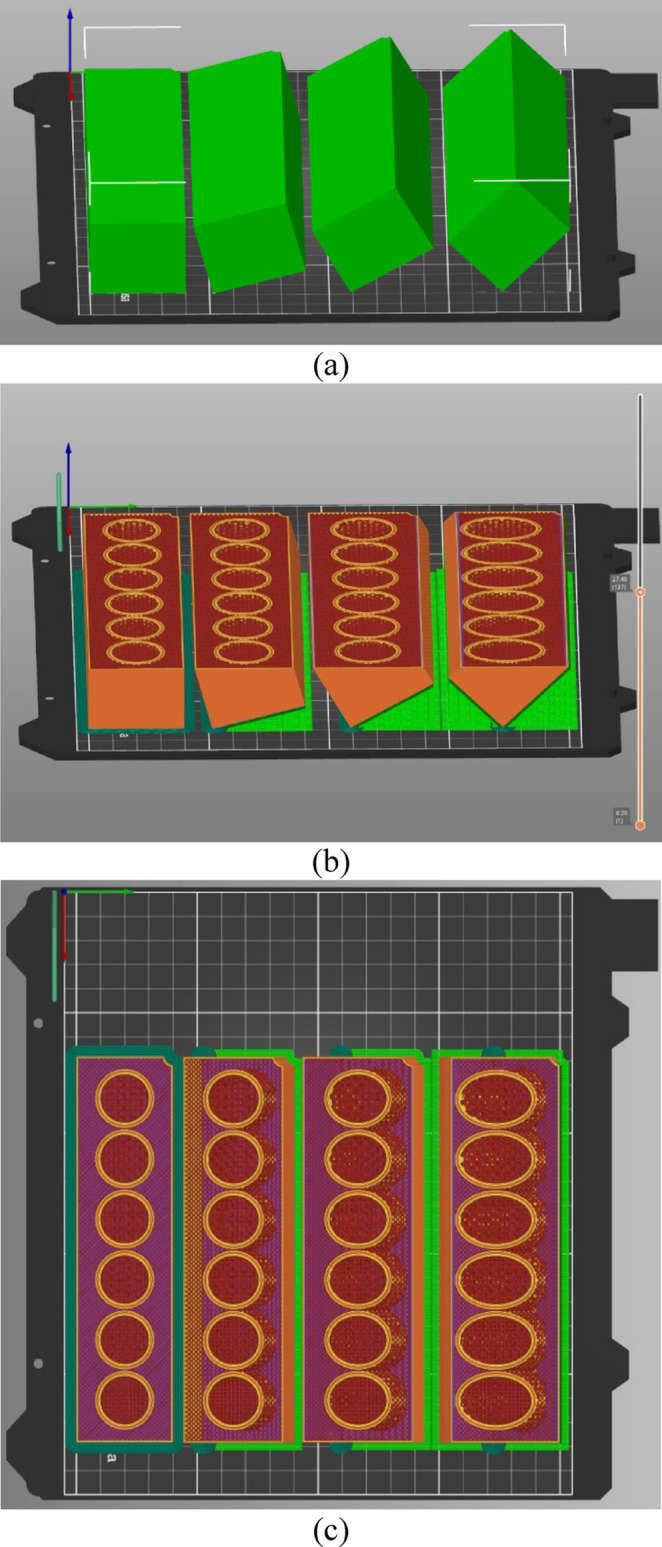
3D printed setup for the surrogate with 3D Honeycomb infill and densities ranging from 17 to 27% with blocks disposed at 0°,15°, 30°, and 45° (**a**), its transverse cross-section shown (**b**), and visualized perpendicularly to the printing axis (**c**)

Titanium cannulated bone screws with 7 mm outer diameter, 20 mm of thread length, and 2.1 mm thread pitch (Rondò, Citieffe, Bologna, Italy) were inserted into pre-drilled holes spaced 25 mm apart in bone surrogate blocks. The surrogates measured 32 mm × 180 mm × 40 mm and were prepared with 3.5 mm pilot holes, matching the screw core diameter to ensure consistent thread engagement across conditions. Two types of bone surrogate material were used: 3D printed and ASTM F-1839-08 polyurethane foams with densities of 10, 15, and 20 PCF (Sawbone, Vashon Island, Washington), corresponding to osteoporotic, normal, and higher than normal bone densities, respectively, as indicated in existing literature [45, 46]. The 3D printed surrogates were modeled in Autodesk Fusion 360 (Autodesk, San Francisco, CA) and printed using Polylactic Acid filament with tensile modulus of 2.3 GPa ± 0.1 and yield strength of 51 MPa ± 3 (Prusa filament, Beige, PLA) on a Prusa MK4 (Prusa Research, Prague, Czech Republic). Infill patterns consisted of 3D Honeycomb (Fig. 1a) and Gyroid (Fig. 1b) [47] structures, printed at densities ranging from 5 to 27% in 2% increments. Infill orientation was obtained by printing the surrogates at the desired inclination of 0,15, 30, and 45 degrees of inclination (Fig. 2). Printing parameters included a layer height of 0.2 mm, a top shell thickness of 1 mm, and a bottom shell thickness of 0.5 mm.

In literature, the use of three repetitions is found in several biomechanical studies on screws torque evaluation, some of which have been performed for medial malleolar fractures [48], for push-out in screw place locking used in internal fixation, and for syndesmosis injury repair [49]. Therefore, our study included 3 testing repetitions for each of the 12 infill densities and 4 inclinations for both patterns, totaling 288 experiments on the printed surrogates [50].

## Mechanical testing

**Fig. 3:**
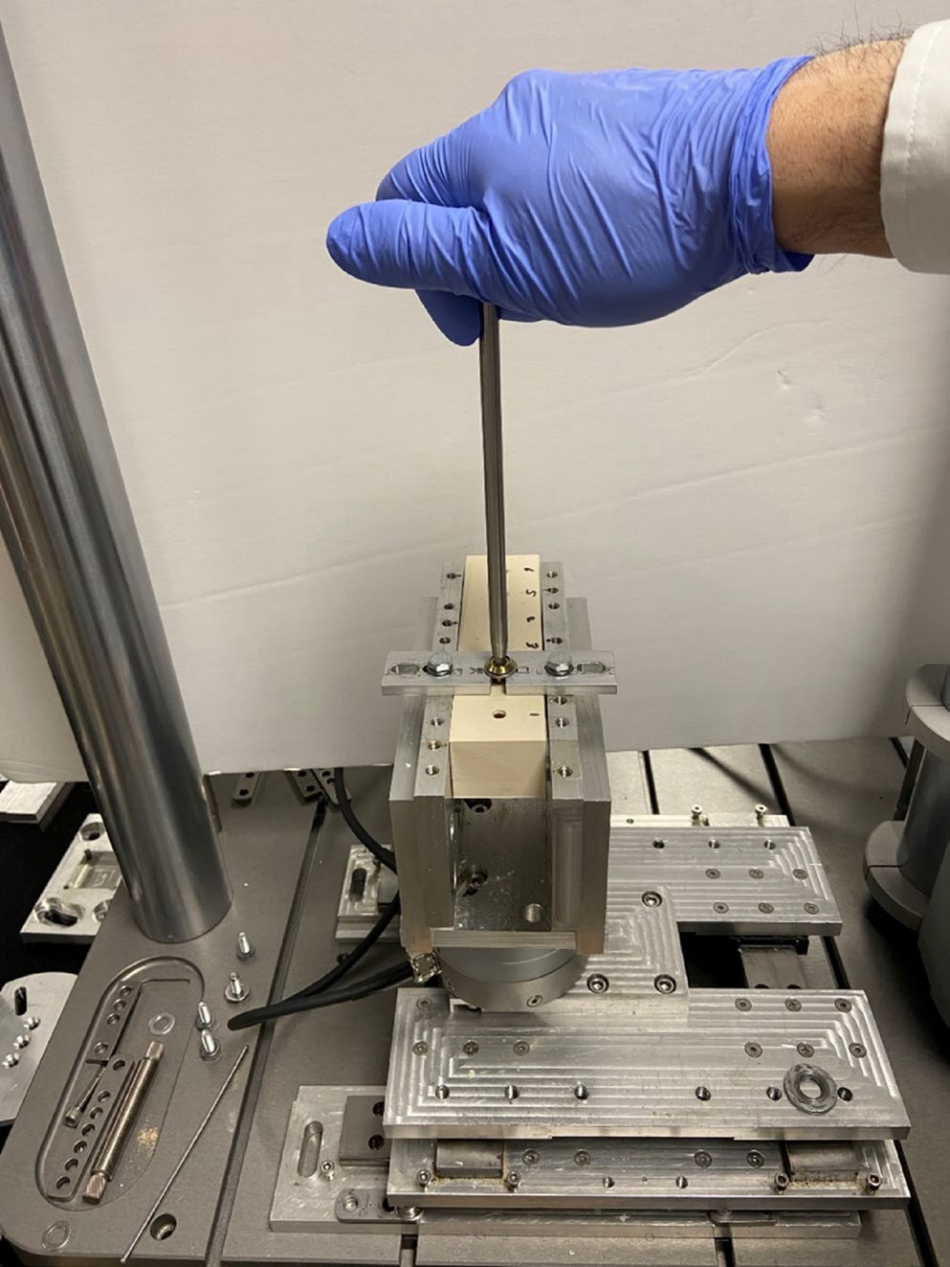
Experimental setup used to measure the stripping torque, while the screw is kept in its axial position by the rigid bar the surrogates are free to move upward without rotation during the screwing, the torque sensor is shown at the bottom of the apparatus

Each surrogate block was mounted in a custom-built fixture designed to constrain rotational motion while allowing free axial translation. This fixture was secured to an Instron E3000 bi-axial testing machine (Instron E3000, Norwood, MA), which housed a torque-sensing load cell calibrated prior to the experiments and mounted below the specimen (Fig. 3), pre-drilled pilot holes ensured consistent screw placement and alignment, eliminating variability from user-driven tracking. All the screws were manually inserted in vertical orientation by the dominant hand of the senior investigator non-surgeon wearing a single type of single layer nitrile gloves. Screws were used in order of increasing surrogate density, and each screw was cleaned and visually inspected for damage after each insertion, discarded if damaged, and not used for more than six repetitions [51].

Torque data were acquired at 100 Hz continuously during each insertion. Screws were advanced by hand without interruptions using a cannulated driver until the moment of mechanical stripping. Stripping was defined as the peak torque immediately preceding the sudden drop in resistance, which corresponds to the screw thread disengaging from the surrounding material. This value was recorded as the ST. No further seating or compression was performed after stripping occurred.

Three independent repetitions were performed for each unique test conditions, defined by a specific combination of infill pattern (Gyroid or 3D Honeycomb), infill density (5 to 27% in 2% increments), and insertion angle (0 °C, 15 °C, 30 °C, 45 °C). The average ST value across these repetitions was used for statistical analysis.

### Statistical analysis

The relationships between stripping torques and infill densities were evaluated through linear regression. This was done by fitting a trendline to the data, applying the method of least squares to determine the best-fitting line that minimizes the sum of squared residuals between the observed and the predicted values. From the trendline equations derived from the dataset, idealized equations were formulated for both 3D Honeycomb and Gyroid infills. Similarly, by plotting the observed ST for standard-weight ASTM polyurethane foams and fitting a trendline, an idealized equation was generated to estimate the ST for these materials. By treating the estimated ST values from the 3D printed surrogates as equivalent to those of ASTM foam, conversion equations were then generated to translate the 3D infill densities into their corresponding ASTM foam densities. Additionally, multivariate linear regression was used to assess for differences in ST when comparing infill densities and screw insertion angles. Separate regression models were calculated using the Gyroid and 3D Honeycomb infill patterns. A pairwise T-Test was performed comparing the insertion angles 15 °C, 30 °C, and 45 °C to the 0 °C angle of insertion to detect any differences in ST caused by the insertion angle. All the analyses were performed with a significance level of 0.05.

## Results

### Screw insertion angle 0 °C (aligned with printing direction)

**Fig. 4:**
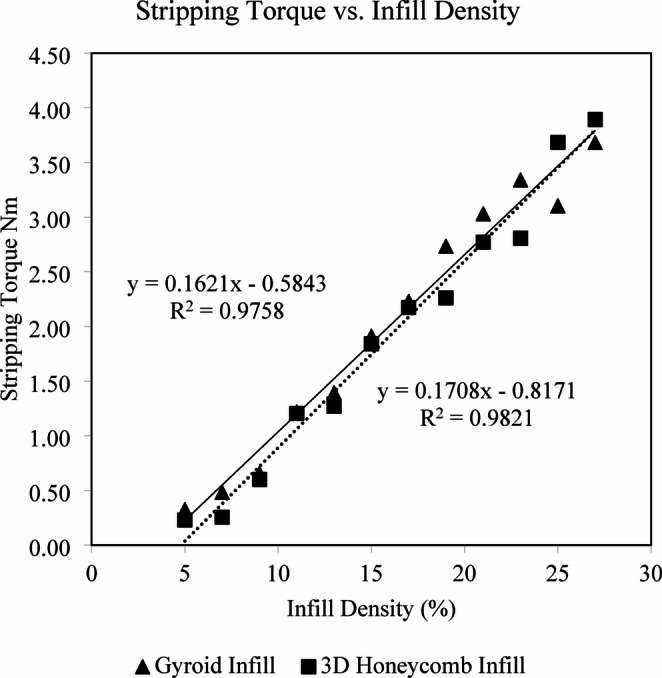
Experimentally obtained Stripping Torque (ST) measured for each of two 3D printed surrogate infill types (Gyroid, 3D Honeycomb) at varying infill densities (%)

**Fig. 5:**
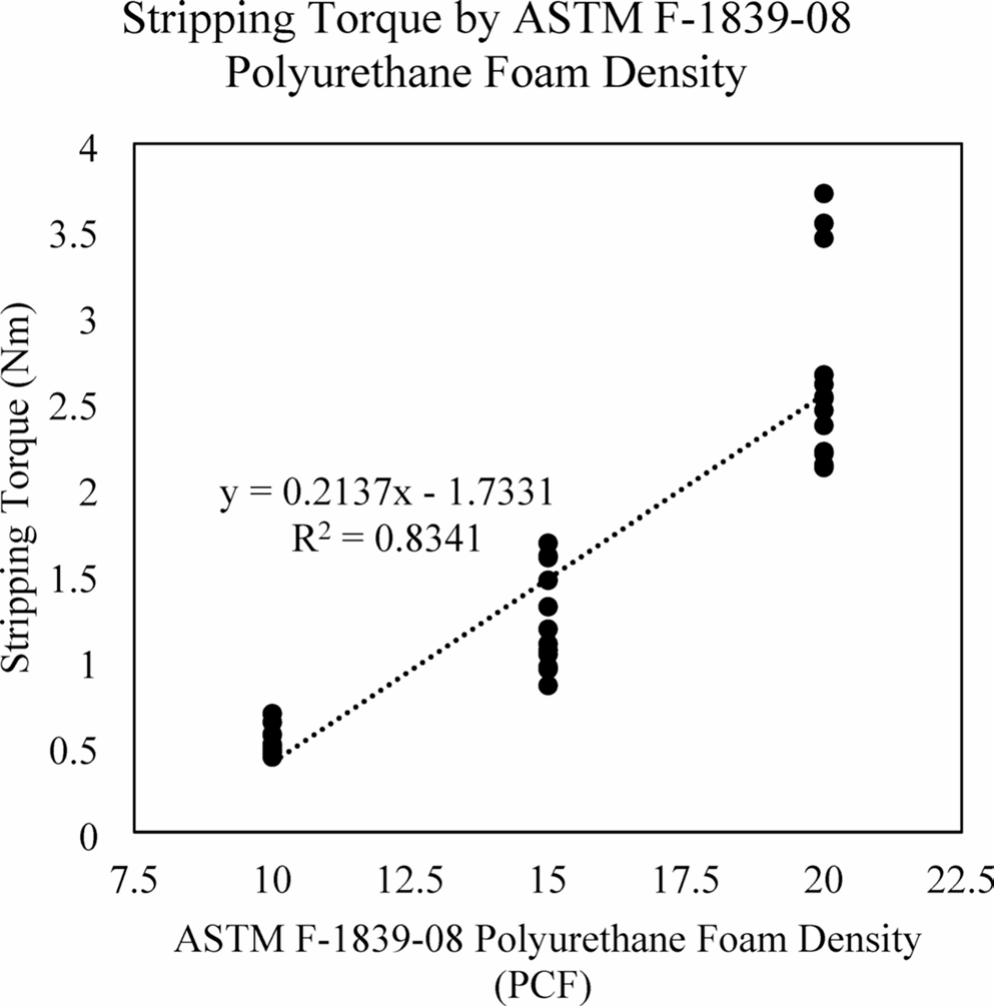
Stripping torque (ST) in Nm at varying ASTM F-1839-08 foam densities (PCF)

Screws were successfully inserted, and torque was applied until the screws were stripped and lost seating in all the 288 prepared specimens without hardware damage. The 3D Honeycomb infill ST ranged from 0.23 Nm ± 0.02 for 5% density to 3.89 Nm ± 0.14 for 27% density infill (Table 1). The ST values found for the Gyroid infill were also monotonically increasing and ranged from 0.32 Nm ± 0.06 to 3.68 Nm ± 0.46 for 5% and 27% infill, respectively (Table 1). A highly linear correlation was found between infill density and ST, R^2^ = 0.98 (*p* < 0.01) and 0.98 (*p* < 0.01) for 3D Honeycomb and Gyroid infill patterns respectively (Fig. 4). For Gyroid and 3D Honeycomb patterns, the ST could be estimated using the idealized equations ST_gyroid_ = 0.16ρ_gyroid_ − 0.58 and ST_3Dhoneycomb_ = 0.17ρ_3Dhoneycomb_ – 0.82, respectively, where ST is expressed in Nm and ρ = infill density (%). In the ASTM F-1839-08 foams we have found ST values that varied in relation to the foam density (*p* < 0.01) and ranged from 0.52 Nm ± 0.08 for the block in 10 PCF, to 1.23 Nm ± 0.29 for 15 PCF, and 2.65 Nm ± 0.55 for the 20 PCF (Fig. 5). The ASTM F-1839-08 foams generated ST values that were also highly correlated with foam density, shown in Fig. 5, R^2^ = 0.83 (*p* < 0.01). The ST from these foams could be estimated using the equation ST_foam_ = 0.21ρ_foam_ – 1.73, where ρ_foam_ = ASTM foam density (Lbs/Cu.Ft). From the idealized equations provided above for each of the infill patterns, as well as that used to describe the estimated ST observed using standard weights of ASTM foam, by treating estimated ST as equivalent, the equation ρ_foam_ = (0.16 ρ_gyroid_ + 1.15)/0.21 can be used to convert Gyroid infill density (%) to the equivalent estimated ASTM foam density. The equation ρ_foam_ = (0.17 ρ_3Dhoneycomb_ + 0.91)/0.21 can be used to convert 3D Honeycomb infill density (%) to the equivalent estimated ASTM foam density. The estimated equivalent ASTM F-1839-08 foam density values that would generate a matching ST value for 10 PCF foams would be a 3D Honeycomb infill pattern with a 7% density and for a Gyroid infill pattern a density of 5.9%, each of the 3D printed infill densities are shown in Table 2.

### Screw insertion at variable angles (not in line with printing direction)

As indicated in Table 3, for the Gyroid pattern, at 5% infill, the ST showed values of 0.32 Nm ± 0.06, 0.26 Nm ± 0.05, 0.36 Nm ± 0.03, and 0.23 Nm ± 0.05 for insertion angles of 0°, 15°, 30°, and 45°, respectively. These values increased at 27% infill to 3.68 Nm ± 0.46, 3.29 Nm ± 0.56, 3.46 Nm ± 0.30, and 3.40 Nm ± 0.54. No significant difference in ST was observed when inserting the screw into the Gyroid pattern at angles of 15° (*p* = 0.69), 30° (*p* = 0.86), or 45° (*p* = 0.73) compared to insertion at 0°.

**Fig. 6:**
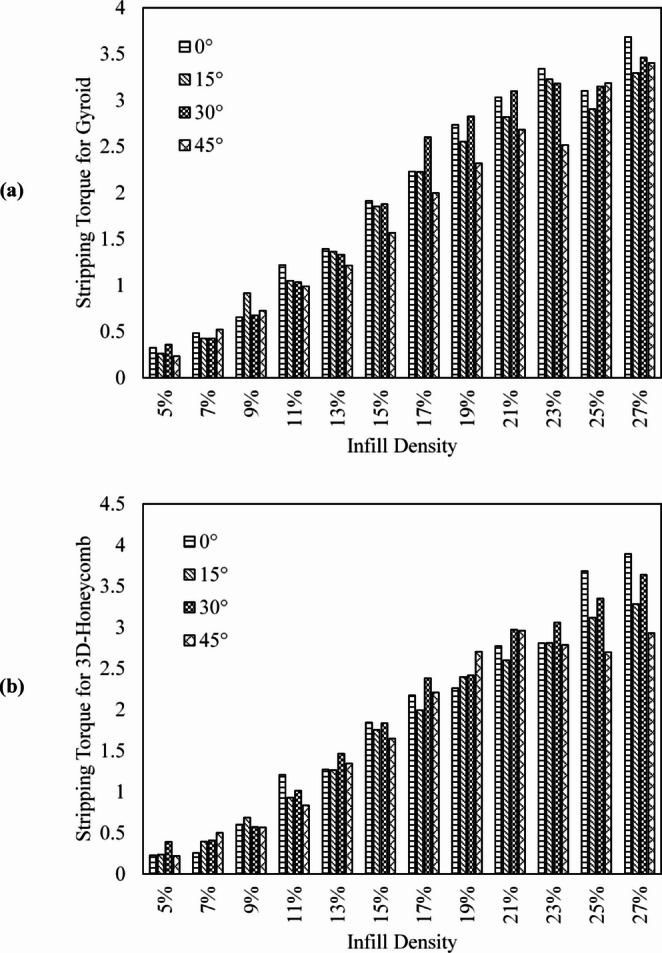
Mean values of experimentally determined stripping torque (Nm), calculated for each group of repetitions, as a function of infill density (%) and angle of insertion (degrees), for Gyroid (**a**) and 3D Honeycomb (**b**) infill patterns

Insertion of screws into the 3D Honeycomb pattern for the inclinations of 0°, 15°, 30°, and 45° resulted in ST of 0.23 Nm ± 0.02, 0.24 Nm ± 0.04, 0.39 Nm ± 0.13, and 0.22 Nm ± 0.02 for the 5% infill and increased to values of 3.89 Nm ± 0.14, 3.28 Nm ± 0.30, 3.64 Nm ± 0.48, and 2.93 Nm ± 0.13 for the 27% infill. Among all the infill densities, differences in ST where not found at 15 degrees (*p* = 0.89), 30 degrees (*p* = 0.95), and 45 degrees (*p* = 0.66, Fig. 6).

## Discussion

In orthopedics surgery, poorly placed or stripped screws put fracture fixation at risk of premature failure [52]. Training of screw fixation is conducted via a trial-and-error process in which trainees tighten screws, then overtighten to cause intentional stripping to give immediate tactile feedback that enhances the trainees’ tactile perception [19]. 3D printed surrogates have been implemented and proven to be effective teaching models in orthopaedic, spine, and dental surgery [41–45]. These models can be customized using medical imaging to simulate unique anatomical features and pathological states that cannot be replicated otherwise [53–55]. One such feature that is not well appreciated or easily replicated with current “sawbones” models is the difference between cortical and trabecular bone, which are of varying densities, and can change screw insertion dynamics greatly [56, 57]. The ability to replicate these differences reliably would enhance current 3D printable training models, as would the ability to replicate metaphyseal versus diaphyseal bone, as these demonstrate varying screw stripping characteristics [58].

To our knowledge, this is the first study to establish a quantitative relationship between 3D printing infill density and ASTM foam density based on stripping torque.

The main finding of the study is a linear relationship between ST and infill density (R^2^ = 0.98; *p* < 0.01), enabling the creation of bone surrogates with predictable and adjustable mechanical properties. This characteristic allows for the creation and sharing of training scenarios for standardized trainee performance evaluation. In the current study, we tested the bone surrogates with partially threaded compression screws commonly used in orthopaedic surgery [59]. Headless compression screws are applied throughout the body by surgeons, and the results that we find in this study could conceivably extend to use of 3D printed surrogates with fully threaded, or other screw configurations [60–62]. The linear relationship we have identified in the current study is analogous to the relationship found by Burkhart et al. during insertion of pedicle screws into spine models [40]. Even if they did not account for the relative inclination between screw insertion and infill direction, using the same Gyroid infill pattern of the current study, have concluded that the 3D printed models were more realistic than standard polyurethane surrogates. The ST found for our 3D printed models was highly correlated to the torque found for the ASTM F-1839-08 polyurethane foam proving that the latter can be replaced in training scenarios already proposed for the foams [46, 63].

In some clinical scenarios, the screw insertion angle is known. For example, regarding femoral neck fractures, surgeons aim for 3 screws inserted in an inverted triangle configuration [64]. For many other situations, insertion angle is determined on a case-by-case basis such as in trauma, since the screw is aimed to be perpendicular to the fracture line that is stochastically determined on the bone segments [65, 66]. Given the variability in bone density across anatomical regions in terms of architecture and density, this characteristic of the 3D-printed surrogates requires site-specific testing to evaluate bone equivalency. The current study is limited to establish equivalency with foam surrogates. In terms of insertion angle, we considered only inclinations in 15-degree increments that were previously proposed for a biomechanical study on the optimum insertion angle for screw-in-anchors although other angles could be considered [67]. In the current study, pairwise* T*-test showed an insignificant difference in ST for insertion angles up to 45 degrees for both Gyroid and 3D Honeycomb infill patterns. This characteristic, found in these two patterns for angles up to 45 degrees, allows great discretion in setting the screw insertion direction and in orienting parts for the 3D printing process.

Manual screw insertion introduces the potential for operator-dependent variability. In this study we did not control or measure screw insertion speed and axial force that are known to influence torque measurements. To mitigate such limitation, all screws were inserted by a single experienced operator using a standardized technique that included consistency of elements previously considered by Fletcher et al. (2020) such as, single type of gloves, use of dominant hand, vertical orientation of the screws, and operator blindness to torque [68]. This approach follows prior studies that intentionally limited insertion to a single operator to reduce inter-user variability and enhance reliability [58, 69, 70]. Examples include studies evaluating maximum screw torque at the femoral diaphysis [58], implant stability in polyurethane blocks [69], and biomechanical comparisons of screw systems [70]. Although this methodology improves internal consistency, it may reduce the generalizability of findings across users with varying skill levels. Future investigations should assess the effects of inter-user variability on ST and the reproducibility of training outcomes.

Another limitation of the current study is the exclusive use of cannulated, partially threaded compression screws with a fixed 20 mm threaded length. Cannulated screws were selected because they are commonly used in orthopaedic trauma for the fixation of a wide range of fracture patterns and bone reconstructions [71, 72]. The central cannulation allows screw placement over a guidewire, improving accuracy and facilitating optimal screw positioning in accordance with the principles of interfragmentary screw fixation [73]. Importantly, ST is governed primarily by the interaction between the screw threads and the surrounding material, therefore, the presence of a central cannulation in unlikely to substantially influence stripping behavior [74]. Partially threaded lag screws are frequently used to achieve interfragmentary compression in cancellous bone, which requires the threaded portion to lie completely beyond the fracture line [75, 76]. In the present study, a solid surrogate was used without fracture interface, therefore, interfragmentary compression was not a functional consideration. Thus, a 20 mm threaded length was arbitrarily selected based on commercially available screw designs. The screw itself was chosen such that its total length was fully contained within the surrogate material. This approach ensured consistent thread engagement and minimized boundary effects across all tests [14, 77]. These design choices improved internal consistency; however, the findings may not be directly generalizable to fully threaded screws or alternative screw configurations. Nevertheless, the linear relationship identified between ST and infill density is expected to extend to other screw designs, a hypothesis that warrants further investigation.

Although the overall cost of surrogate models depends on institutional factors such as purchasing agreements, access to bulk pricing, and in-house fabrication capabilities, a general comparison offers practical insight. In our study, commercially available ASTM F-1839-08 polyurethane foam blocks (Sawbones; 130 mm × 180 mm × 40 mm) were used and sectioned into four strips each, yielding a cost per training block of $4.37 for 10 PCF and $5.88 for 20 PCF foam densities [78, 79]. Using idealized regression equations derived in our results, the estimated equivalent 3D printed infill densities for these foam types were 6 and 19% for gyroid infill and 7% and 19% for 3D honeycomb infill, respectively (Table 2). The corresponding gyroid models cost approximately $1.65 for 6% infill (print time: 3 h 27 min) and $2.87 for 19% infill (6 h 53 min). For 3D honeycomb models, costs were $1.73 for 7% infill (3 h 42 min) and $3.12 for 19% infill (8 h 20 min). These figures do not include printer purchasing costs or machine running costs; however, their amortization is feasible given the margin between material costs and those of standard polyurethane foam blocks. While material cost alone does not determine overall cost-effectiveness, it is an important consideration when evaluating training solutions. In addition to offering lower material costs, 3D printed models offer several other advantages, including the ability to generate patient specific, anatomically identical models from imaging data (CT scans) [80, 81]. These models reduce the need for cadaveric or animal material, dedicated lab space, and personnel. Furthermore, they can be stored digitally, reproduced on demand, used on site, recycled to reduce the carbon footprint, and shared across training sites with consistency—capabilities not possible with conventional foam blocks. This flexibility enhances scalability and standardization, making 3D-printed surrogates a practical and innovative option for surgical training environments. The main limitation of the study should be found in the number of repetitions that were established in three. This approach aligns with common practice in mechanical testing literature to account for within-condition variability and ensure reproducibility [82–84].

One limitation of the current study is the fact that our torque measures were compared to foam bone surrogates and not human bones. This was chosen because ASTM F-1839-08 polyurethane foam is the most widely used bone surrogate material for mechanical testing and instrumentation in orthopaedics, having properties that are analogous to human bones [53, 85]. In testing the polyurethane foams, we recorded the nominal density without measuring the actual density of the tested blocks, which are know to show strong variability in mechanical characteristics [86–90], that according to the manufacturer, can vary by ± 10% within a single batch. In the current study, additional variation arose from using foams from different batches stored in the lab. While this introduced variability in ST, it further supports the use of 3D-printed surrogates, which allow precise control of infill density and can be produced on demand. A further limitation is that we neither controlled nor measured the insertion rate, as screws were inserted manually.

An additional limitation is the consideration of only two infill patterns. Other infills are available but less utilized, often proprietary, and not available in most 3D printer software [91–93]. Another element of limitation is the infill density value that we have considered limited to 27%. This peak value was chosen because it has been shown, for femurs, to replicate mechanical behavior closer to bone than polyurethane surrogates [94]. A further limitation of this study is the use of three repetitions per test condition. While this is consistent with common practice in mechanical surrogate testing, it limits the precision of within-condition variance estimates and may reduce sensitivity to detect small angle-dependent differences in ST. Future studies with larger per-condition sample sizes would allow more precise characterization of angle-dependent variability, particularly at higher densities where greater absolute variance was observed.

Moreover, in generating the 3D-printed surrogates, the infill density was imposed without measuring the resultant geometrical characteristics of the 3D-printed infill or their influence on the stripping torque. This specific interaction should be evaluated in future studies.

In conclusion, while potential educational and clinical benefits require further studies, we have found that the 3D printed PLA surrogates with 3D Honeycomb and Gyroid infills and density percentages up to 27% are mechanically comparable to ASTM F-1839-08 polyurethane foam for ST in screw insertion training. Furthermore, the direction of screw insertion into these 3D printed models can be considered independent from the 3D printed direction for inclinations up to 45 degrees, simplifying the printing process and widening the field of application to training scenarios with unset screw insertion direction. Combining variability in screw insertion angles with the ability to replicate a variety of bone densities, as well as various pathologies, would allow for the ability to produce bone surrogate with a single print that provides a more realistic option than polyurethane foams.

**Table 1:** Stripping torque (ST) values for screw insertions into two infill patterns averaged across three trials at an angle of 0° (perpendicular to the printing axis)

% Infill density	Stripping torque (Nm ± SD)	
	Gyroid pattern	3D Honeycomb pattern
5	0.32 ± 0.06	0.23 ± 0.02
7	0.48 ± 0.09	0.26 ± 0.05
9	0.66 ± 0.08	0.6 ± 0.11
11	1.22 ± 0.01	1.2 ± 0.18
13	1.39 ± 0.07	1.27 ± 0.11
15	1.91 ± 0.25	1.84 ± 0.30
17	2.23 ± 0.12	2.17 ± 0.29
19	2.73 ± 0.10	2.26 ± 0.25
21	3.03 ± 0.38	2.77 ± 0.19
23	3.34 ± 0.22	2.81 ± 0.49
25	3.10 ± 0.61	3.68 ± 0.47
27	3.68 ± 0.46	3.89 ± 0.14

**Table 2:** Equivalent ASTM F-1839-08 polyurethane foam densities for different percentage densities of Gyroid and 3D Honeycomb infills given by the idealized equations: ρfoam = (0.16ρgyroid + 1.15)/0.21; ρfoam = (0.17ρhoney + 0.91)/0.21

% Infill Density	Equivalent ASTM F-1839-08 polyurethane foam densities	
	Gyroid (PCF)	3D Honeycomb (PCF)
5	9	8
7	11	10
9	12	12
11	14	13
13	15	15
15	17	16
17	18	18
19	20	20
21	21	21
23	23	23
25	24	25
27	26	26

**Table 3:** Average values and standard deviations across repetitions by infill density and insertion angle, with p-values calculated relative to 0° insertion

	% Infill density	0°	15°	30°	45°
Gyroid	5	0.32 ± 0.06	0.26 ± 0.05	0.36 ± 0.03	0.23 ± 0.05
	7	0.48 ± 0.09	0.42 ± 0.10	0.42 ± 0.05	0.52 ± 0.12
	9	0.66 ± 0.08	0.91 ± 0.38	0.67 ± 0.10	0.73 ± 0.34
	11	1.22 ± 0.01	1.05 ± 0.22	1.03 ± 0.19	0.99 ± 0.28
	13	1.39 ± 0.07	1.36 ± 0.58	1.33 ± 0.44	1.21 ± 0.30
	15	1.91 ± 0.25	1.85 ± 0.29	1.88 ± 0.32	1.57 ± 0.11
	17	2.23 ± 0.12	2.22 ± 0.23	2.60 ± 0.33	2.00 ± 0.08
	19	2.73 ± 0.10	2.55 ± 0.44	2.83 ± 0.17	2.32 ± 0.20
	21	3.03 ± 0.38	2.82 ± 0.18	3.10 ± 0.40	2.68 ± 0.05
	23	3.34 ± 0.22	3.23 ± 0.11	3.18 ± 0.49	2.52 ± 0.39
	25	3.10 ± 0.61	2.90 ± 0.40	3.15 ± 0.64	3.19 ± 0.58
	27	3.68 ± 0.46	3.29 ± 0.56	3.46 ± 0.30	3.40 ± 0.54
	*p*-value	–	0.69	0.86	0.73
3D Honeycomb	5	0.23 ± 0.02	0.24 ± 0.04	0.39 ± 0.13	0.22 ± 0.02
	7	0.26 ± 0.05	0.39 ± 0.13	0.41 ± 0.05	0.50 ± 0.15
	9	0.60 ± 0.11	0.69 ± 0.10	0.57 ± 0.15	0.57 ± 0.11
	11	1.20 ± 0.18	0.93 ± 0.06	1.01 ± 0.20	0.83 ± 0.16
	13	1.27 ± 0.11	1.26 ± 0.07	1.46 ± 0.22	1.34 ± 0.13
	15	1.84 ± 0.29	1.75 ± 0.12	1.83 ± 0.39	1.65 ± 0.07
	17	2.17 ± 0.30	1.99 ± 0.28	2.38 ± 0.32	2.21 ± 0.19
	19	2.26 ± 0.25	2.40 ± 0.21	2.42 ± 0.42	2.70 ± 0.08
	21	2.77 ± 0.19	2.60 ± 0.48	2.97 ± 0.40	2.96 ± 0.45
	23	2.81 ± 0.49	2.81 ± 0.45	3.06 ± 0.35	2.79 ± 0.66
	25	3.68 ± 0.47	3.12 ± 0.53	3.35 ± 0.36	2.70 ± 0.15
	27	3.89 ± 0.14	3.28 ± 0.30	3.64 ± 0.48	2.93 ± 0.13
	*p*-value	–	0.89	0.95	0.66

## Supplementary Information

Below is the link to the electronic supplementary material.

Supplementary Material 1

## Data Availability

The datasets generated during and/or analyzed during the current study are available from the corresponding author on reasonable request.
